# An Individual Music Intervention for Adults With Intellectual Disabilities and Challenging Behavior: Protocol for a Randomized Controlled Trial

**DOI:** 10.2196/52497

**Published:** 2024-02-07

**Authors:** Gerianne Smeets, Karin Volkers, Erik Scherder, Xavier Moonen

**Affiliations:** 1 Philadelphia Care Foundation Amersfoort Netherlands; 2 Department of Clinical Neuropsychology VU University Amsterdam Amsterdam Netherlands; 3 Research Institute of Child Development and Education University of Amsterdam Amsterdam Netherlands

**Keywords:** music intervention, intellectual disability, challenging behavior, executive functioning, self-esteem, anxiety, depression, randomized controlled trial, RCT, study protocol, well-being

## Abstract

**Background:**

Individuals with intellectual disabilities (ID) are more likely to have problems with executive functioning (EF) and challenging behavior (CB), which are negatively linked to well-being. Among clinical populations, music interventions have been shown to improve various outcome measures, such as CB and EF. Until now, no randomized controlled trials (RCTs) have been conducted to examine the effectiveness of an individual music intervention for adults with ID and CB.

**Objective:**

The study aims to identify the effect and feasibility of an individual music intervention compared with care-as-usual for people with ID and CB.

**Methods:**

In this study, a 2-group RCT with a pretest, posttest, and follow-up assessment after 8 weeks is presented. Participants of the music intervention condition will receive 16 individual music sessions within 8 to 10 weeks. The music intervention will be guided by a manual for music workers, in which every session will have a different focus (introduction, emotions, different EF, and end performance). Participants receiving care as usual will function as a control group. After the research is finished, they will be offered a budget, which they can spend on musical activities or musical instruments as they wish. Assessments will include caregiver rating scales and self-report questionnaires and tests, which will assess outcome measures of CB, well-being, depression, anxiety, self-esteem, and 4 domains of EF. A process evaluation will be conducted after the completion of the study, which entails the analysis of data on multiple aspects of the intervention and the study overall.

**Results:**

Enrollment commenced in July 2021, and data collection ended in May 2023. A total of 97 participants were recruited, with 44 participants allocated to the intervention group and 53 allocated to the control group. Data will be analyzed after this protocol has been accepted for publication.

**Conclusions:**

Because there are currently no published RCTs of an individual music intervention for adults with ID and CB, this study will provide insight into the effectiveness and experiences of an individual music intervention for this target group.

**Trial Registration:**

International Clinical Trials Registry Platform NL8482; http://tinyurl.com/4565s5pd

**International Registered Report Identifier (IRRID):**

DERR1-10.2196/52497

## Introduction

### Background

Intellectual disability (ID) is characterized by significant limitations in both intellectual functioning and adaptive abilities, such as conceptual, social, and practical skills. ID originates before the age of 18 years and can be categorized as mild, moderate, or profound [1]. Besides having a lower IQ and problems with adaptive functioning, people with ID are more likely to develop mental health problems, for example, affective and anxiety disorders and challenging behavior (CB) [2,3]. Mental health and CB are negatively linked to quality of life (QoL) [4].

People with ID not only have a higher chance of developing mental health problems and CB, but there is evidence that their executive functioning (EF) is also impacted [5]. EF refers to the set of abilities involved in planning, self-monitoring, and purposive action, which are “at the heart of all socially useful, personally enhancing, constructive, and creative activities” ([6], p. 281). There is broad consensus that there are 3 core EFs: inhibition, working memory, and cognitive flexibility [7,8]. Some studies in the ID field use a broader definition that includes attention [9,10], planning, and categorization [9]. A few studies suggest that regarding EF, people with ID perform at levels commensurate with their mental age [11,12]. Mental age can be calculated from raw scores on intelligence tests: mental age = (IQ score × chronological age) / 100 [13].

Poorer performance on EF tests is linked to more CB, especially externalizing behavior problems, among children, adolescents, and adults with ID. This holds for tests that measure inhibition [14], working memory [15], and cognitive flexibility [16]. Because CB has a major impact on the lives of people with ID and their caregivers, attempts to help diminish CB cover a wide range of different approaches, from environmental approaches [17] and support staff training [18-20], to pharmacological interventions [21]. In 2 meta-analyses, effects were found in reducing CB among people with ID for biological and nonpharmacological (such as psychotherapeutic and contextual) interventions [22,23]. Another example of such a nonpharmacological intervention is music therapy, which a recent review found to have a positive effect on CB, anxiety, self-esteem, management of emotions, cognitive measures, and QoL of people with ID [24]. However, this review concluded that most of these studies had several shortcomings, such as no control group, small sample sizes, lack of follow-up measurements, and no use of self-reports.

Several empirically valid studies have been carried out with participants who have other cognitive or communication disabilities, such as dementia, acquired brain injury, or autism spectrum disorder [25]. In these studies, beneficial effects of music interventions on various outcomes were found, such as self-esteem [26], anxiety [26-29], CB [27], mood [28,30,31], QoL [30,31], and EF (ie, working memory, attention, and EF in general) [30]. However, even in these well-researched fields, there seems to be a paucity of high-quality empirical studies [32,33], which emphasizes the need for more rigorous randomized controlled trials (RCTs) with sufficiently large sample sizes studying the different effects of music interventions.

Although music interventions seem effective on CB, anxiety, mood, self-esteem, management of emotions, cognitive measures, and QoL, it is worth noting that the type of music interventions used can vary widely, depending on the goal and the characteristics of the participant. Some interventions are group sessions, whereas others are individual music sessions that can be tailored more to a participant’s individual needs and that show tangentially higher effects, for instance, on agitation in persons with dementia [34]. Some interventions include music therapy (which involves a trained professional using music to address specific therapeutic goals), music listening, or general music-based interventions [35]. It is not uncommon to follow a guideline, with specified principles and procedures of the music intervention, while still leaving enough flexibility to tailor the intervention to the characteristics and needs of the participant and the specific requirements of the situation [33]. Music interventions range from daily to weekly, lasting 10 to 60 minutes, and can have “passive” (listening) or “active” (making music or sounds together) components [33,36]. Active music interventions generally provide greater individual benefits than passive music engagement among older people [37,38], people with autism spectrum disorder [36], and in the general population [39].

Overall, evidence suggests that active music participation can enhance one’s emotional, psychological, and social well-being and even increases EF (eg, working memory and attention) in specific clinical populations; however, to the best of our knowledge, no RCTs are available assessing the effectiveness of an individual music intervention for adults with ID, let alone for adults with ID and CB. In this protocol paper, the design and rationale of an RCT are described, in which a manual for an individual active music intervention for adults with ID and CB is presented, matched with persons with ID and CB who receive care as usual (CAU) functioning as a control group.

### Objectives

#### Primary Objective

The primary objective of the proposed RCT is to evaluate the effect of receiving 16 individual active music sessions compared with CAU in adults with a mild or moderate ID with CB on various outcome measures such as CB, well-being, depression, anxiety, self-esteem, and 4 domains of EF.

#### Secondary Objective

A secondary goal of this study is to evaluate the implementation, impact, and context of the individual music intervention with input from participants, caregivers, and music workers.

## Methods

### Study Design

**Figure 1:**
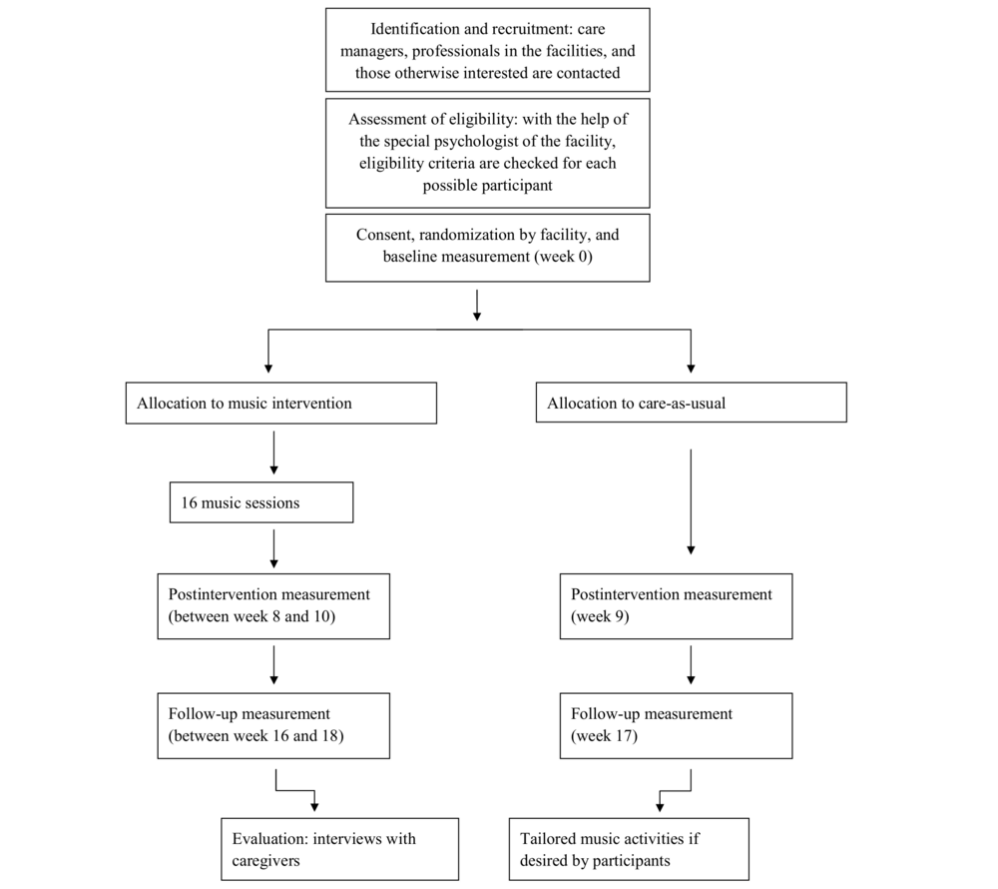
Study flowchart.

This study is an RCT with a pretest (T1), posttest (T2, 9 weeks after T1, unless the last intervention session was not yet completed; posttest was postponed by 1 or 2 weeks until the week after the final session), and follow-up assessment (T3, 8 weeks after T2), including 2 groups of individual participants (see Figure 1). Participants belonging to the music condition will receive 16 individual music sessions within 8 to 10 weeks, and participants belonging to the control condition will receive (individual) CAU. Randomization will be performed at a facility level for 3 reasons: first, to prevent possible interference between participants belonging to both groups in a facility; second, it is deemed unethical and difficult to explain to people with ID who are assessed to the control condition that they have to wait for the music sessions, whereas fellow residents already receive them; finally, it can also promote recruitment [17]. For randomization, a computerized random number generator will be used. There is no maximum number of participants per facility. Blinding is not possible because it is clear to the caregivers, participants, and research assistants which type of intervention a participant receives. The independent research assistants are also not blinded because the participants of the music intervention have to fill in an extra questionnaire at the second and third assessment.

### Study Population

Participants will be included based on 4 criteria and 3 exclusion criteria. The inclusion criteria are as follows: (1) participants must have a mild or moderate ID, (2) they are 18 years or older, (3) they show either internalizing or externalizing CB, and (4) they are mentally competent to give consent to participate in this research. The exclusion criteria are as follows: (1) inability to participate in an intervention for at least 1 hour; (2) a hearing impairment that cannot be corrected with a hearing aid; and (3) a serious medical condition that limits participation, such as dementia. Already being involved in prior musical activities is not an exclusion criterion.

### Procedure

Participants will be recruited from residential facilities for people with ID of the Philadelphia Care Foundation throughout the Netherlands. Because participating in this study requires time and effort of their staff, care managers of facilities serving adult residents with mild to moderate ID are contacted about their willingness to participate in the study. Managers with interest will receive an information letter explaining the research aims and responsibilities of all parties involved. Next, the special psychologist of the facility will check which residents are eligible for inclusion, who then will receive an information letter and an informed consent form in a Dutch easy-to-read format [40]. Caregivers will be asked to read the letter together with potential participants and help them to fully understand the text, if needed. Legal representatives of the eligible residents will also receive an information letter and an informed consent form, whereas nonlegal representatives of the eligible residents will only receive an information letter. Residents will only become participants in the study if (both) informed consent forms are signed. Subsequently, the special psychologist of the facility will be asked for the participant characteristics, which are age, gender, level of ID, syndrome (when applicable), and comorbid psychiatric diagnoses.

After all assessments are completed, participants will receive a compliment postcard from the Philadelphia Care Foundation and a gift card of €15 (US $17.82 when enrollment commenced).

### Intervention

In this RCT, 2 conditions are compared: a music intervention and CAU.

#### Music Intervention Group

The music intervention consists of 16 individual music sessions held twice a week within 8 to 10 weeks. Besides this individual music intervention, regular musical activities that participants already are involved in before the start of this study will continue, as music participation is a central theme of the Philadelphia Care Foundation. The 16 music sessions will be conducted by a music worker, who can be anyone who is able to play an instrument and is enthusiastic and capable to conduct musical sessions with a person with ID. Music workers are not music therapists because the focus is not on therapy but on a general active music intervention. Before the start of the music intervention, the facility in which it will take place will receive a box with an assortment of different kinds of musical instruments, including rhythm instruments (eg, cajon, djembe, and small percussion) and harmony and melody instruments (eg, keyboard and guitar). In general, during the sessions, the music worker adapts the genre or specific pieces of music to the wishes and preferences of the participant. The music intervention will be guided by a training manual for the music workers, in which the frequency, average time per (elements of the) session, and the content of every music session are outlined. The manual is designed based on the input of different advisory groups with professional music coaches, caregivers, and people with ID.

In the manual, intervention procedures are specified, such as the setting, general goals, contents of the music sessions, basic principles of the intervention, and exemplifications. An outline of the music session can be seen in Table 1. Every session will be centered around a different aspect (introduction, emotions, different EF, and end performance), and in the training manual, example exercises are described focusing on every aspect. These examples are provided by advisory groups of professional music coaches and stem from different other manuals [41-43]. Each session will start with a welcome song, after which a listening exercise will take place. Then, a warm-up of the voice and body is scheduled, and subsequently, participants actively engage in music activities by singing or playing an instrument, after which a cool-down and a farewell song are performed. The manual provides a fixed frame for the intervention, but it leaves enough flexibility to tailor the intervention to the characteristics and needs of every participant. Participants will receive a diploma at the final session.

To study for exploratory purposes the impact of the music sessions’ frequency, some participants will receive extra music sessions, resulting in 24 to 27 sessions within the same time period, aiming at approximately 3 sessions a week.

**Table 1:** Outline music session.

Music intervention	Time (minutes)	Attendees
Preliminary meeting	30	Music worker, participant, and caregiver
Music session 1: Welcoming or introduction 2-6: Emotions 7+10: EF^a^ (Inhibition) 8+11: EF (Cognitive flexibility) 9+12: EF (Working memory) 13-15: Working toward the end performance or song 16: End performance or song	60	Music worker and participant
Final meeting	30	Music worker, participant, and caregiver

^a^EF: executive functioning.

#### CAU Group

Participants receiving CAU will function as a control group. CAU includes regular care such as assistance with acts of daily living and day care activities. CAU can include musical activities that participants already performed before the start of this study, as music is a central theme of the Philadelphia Care Foundation. However, new individual musical activities will be postponed for these participants until after the follow-up measurement. Participants receiving CAU will be offered a budget at the end of the study, which they can spend on musical activities or musical instruments to their own wishes, with the help of a musical specialist who works at the Philadelphia Care Foundation.

### Measures

All assessments (T1, T2, and T3) will take place by applying caregiver rating scales and self-report questionnaires and tests and all will be conducted in the same week. Students from several Dutch universities will work as research assistants assisting with the data collection. Research assistants receive theoretical and practical training in assessment through a training program in the test administration protocol. They will administer all irregularities and deviations from this protocol in a study log report. The research assistants will not be involved in the intervention itself. An overview of the outcome measures and the process evaluation can be seen in Table 2.

**Table 2:** Overview outcome measures and process evaluation.

Assessment			Proxy or self-report							Measurement moment								
			Caregiver		Music worker		Person with ID^a^		Each music session			Baseline (T1)		Postintervention (T2)		Follow-up (T3)		After follow-up
**Outcome measures**																		
	Self-reports and tests					✓					✓		✓		✓			
	Proxy questionnaires	✓									✓		✓		✓			
**Evaluation^b^**																		
	Evaluation questionnaire music sessions			✓		✓							✓					
	Follow-up evaluation	✓				✓									✓			
	Self-report with emoticons					✓		✓										
	Log reports music sessions			✓				✓										
	Semistructured interview	✓															✓	

^a^ID: intellectual disabilities.

^b^Only in the music intervention.

### Outcome Measures

#### Challenging Behavior

The Dutch version of the Aberrant Behavior Checklist (ABC) will be used to assess CB, and the form will be filled out by a caregiver [44]. The ABC consists of 58 items, which are rated on a 4-point Likert scale, ranging from 0 (“not at all a problem”) to 3 (“the problem is severe in degree”). The sum of the questions provides a total score (range 0-174), with higher scores indicating more CB. In this study, the cluster structure as suggested by Kaat et al [45] will be used. The first cluster (28 items; α=.96, β=.57) represents behavior that is directed outward (ie, externalizing CB). The second cluster (26 items; α=.93, β=.76) represents behavior that is directed inward (ie, internalizing CB). For externalizing CB, the score ranges from 0 to 84, and for internalizing CB, the score ranges from 0 to 78. The total score on cluster 1 (ie, externalizing CB) will be used to assess externalizing CB, the total score on cluster 2 (ie, internalizing CB) will be used to assess internalizing CB, and the total score on all items of the ABC will be used to assess overall CB.

#### Well-Being

Subjective well-being will be assessed using the Dutch version of the 7-item Personal Well-being Index—Intellectual Disability (PWI-ID) [46]. An example of an item is as follows: “How happy do you feel about the things you have? Like the money you have and the things you own?” Each item is scored on a 3-point Likert scale (0=sad, 1=neither happy nor sad, and 2=happy). Three pictures of colored smileys are used to represent the 3 answers. The pretesting protocol, which screens for acquiescent responding, will not be applied, to diminish the data collection burden for participants. PWI-ID scores range from 7 to 24, with higher scores indicating a better subjective well-being. The PWI-ID seems to be an appropriate measure for people with a mild or upper moderate level of ID [47].

#### Depression and Anxiety

Symptoms of depression and anxiety will be assessed using the Depressed Mood and General Anxiety subscales from the Dutch version of the Anxiety, Depression and Mood Scale [48]. This questionnaire will be completed by the caregiver. The Depressed Mood subscale consists of 13 items, for example, “somber mood,” which are scored on a 4-point Likert scale (0=behavior has not occurred or is not a problem, 1=behavior sometimes occurs or is a slight problem, 2=behavior often occurs or is a relatively large/moderate problem, and 3=behavior occurs a lot or is a severe problem). Depression scores can range from 0 to 39. The General Anxiety subscale of 7 items, for example, “nervous or anxious,” are also scored on this 4-point Likert scale. Anxiety scores range from 0 to 21. For both subscales, a higher score indicates more symptoms. Its reliability and validity are satisfactory to good [49].

#### Self-Esteem

Self-esteem will be assessed using the 5-item Global Self-Worth subscale of the Self-Perception Profile for Adolescents [50]. In this study, the Dutch adaptation of the Self-Perception Profile for Adolescents [51] will be used, with a Cronbach α of >.70 and 1 statement per item [52]. An example of an item is “I am quite happy with myself.” Each item is scored on a 4-point Likert scale (1=completely untrue for me, 2=a little untrue for me, 3=a little true for me, and 4=completely true for me). The total score can range from 5 to 20, and a higher score indicates higher self-worth.

#### EF Tasks

##### Overview

EF will be assessed on a tablet using 3 computer-based games to assess cognitive flexibility, attention, inhibition, and working memory, which were custom-developed in earlier research and are adapted to the needs of individuals with ID, that is, fewer trials, shorter tasks, visual support during test instructions, and attractive stimuli [14,53]. After each game, the research assistant will complete 2 questions about how often the participant needed to be motivated and how often the participant was distracted during the games.

##### Cognitive Flexibility

The game to assess cognitive flexibility is based on the flanker task [54]. The principle of the flanker task is that participants have to respond to a target arrow, which is flanked by distracter arrows. In this study, this task will consist of 5 arrows per trial, with 32 random trials per condition, with 3 conditions. In the first condition, there are 5 green arrows pointing in different directions (left or right). Participants have to ignore the irrelevant stimuli and press the button in the same direction as the green arrow in the middle. In the second condition, the participants are presented with red arrows. Unlike the previous condition, participants have to press the button with the opposite direction as the arrow in the middle. In the third condition, participants are presented with alternating trials of red or green arrows and are requested to press the button with the opposite direction if the arrow in the middle is red and to press the button with the same direction if the arrow in the middle is green. All 3 conditions had a practice round before the official task. The score used to measure cognitive flexibility consists of the correct responses in the third condition (range 0-32). A higher score implies a higher degree of cognitive flexibility.

##### Attention

Attention will be assessed with the first condition of the already mentioned flanker task, where participants have to respond to the green arrow in the middle, regardless of the nature of the flanking distractor items, by pressing the right button for right-faced arrows and the left button for left-faced arrows. Both correct and incorrect (ie, missing or lack of on-time response) responses will be added, standardized as *z* scores, to create a composite total attention score. The incorrect responses will be reversed to create a variable in which a higher score represents better attention.

##### Inhibition

Inhibition will be assessed using two different tasks: (1) the second condition with red arrows of the already mentioned flanker task, which measures interference control [54] and (2) the go or no-go paradigm, which measures the inhibition of a prepotent motor response [55]. The *z* scores on both outcome variables will be added to create a composite total inhibition score for each participant. This new variable will be reversed, so a higher score represents better inhibition skills.

The outcome variable from the flanker task that will be used is the number of faults in the task with only red arrows, representing the inability to suppress an initial response. In this task, participants have to press the arrow corresponding with the opposite direction of the central arrow.

The go or no-go task consists of 2 parts. In the first part, the participant is requested to press the button as soon as a green apple, that is, a “go” stimulus, appears on the screen. In the second part, participants have to tap on the button when the green apple appears, but they have to inhibit their response when a red cross appears through the apple, that is, a “no-go” stimulus. An incorrect answer is given when the participant presses the button, and the green apple appears with a red cross. Both conditions had a practice round before the official task. The outcome measure for the go or no-go task is the total incorrect answers in part 2.

##### Working Memory

To assess the working memory construct, a visuospatial computerized task, based on the Klingberg principles for working memory, will be used [56,57]. In the Klingberg task, participants have to remember the order that a circle shifts over a 4-by-4 grid of open squares. The task consists of 2 conditions that both start with an instruction and a 4-trial practice session before the regular session. The regular session starts with a pattern of 2 circles on adjacent units and gradually increases in length and difficulty with each trial. Each trial consists of 2 patterns of the same length and difficulty. In the first condition, patterns with a green circle are presented to the participants who have to tap on the squares in the grid in the same order afterward (forward version). In the second condition, patterns of red dots are shown which participants have to tap in the reverse order afterward (backward version). When a participant taps a wrong pattern twice on a trial of the same length and difficulty, the task ends. In this study, the average number of total correct trials in the forward and backward task will be used as a measure of working memory [56,58]. A higher score represents better working memory. There is no absolute maximum score; in theory, the task can go on indefinitely.

### Process Evaluation

#### Overview

Because conducting clinical trials in the field of adults with ID and CB seems to be challenging [25], it is also important to share the practical experiences when conducting trials using a process evaluation [59]. For conducting a process evaluation, different frameworks are developed, which all focus on several process evaluation components. For this study, the following frameworks are used: the Medical Research Council guidance [59], process evaluations for cluster-randomized trials of complex interventions as proposed by Grant et al [60], and the RE-AIM (Reach, Effectiveness, Adoption, Implementation, and Maintenance) framework [61,62]. The Medical Research Council guidance and the RE-AIM framework have recently been used in the ID-research field [19,63-65].

#### Evaluation per Music Session

The music worker will ask the participant before and after each music session how he or she feels at that moment: happy, sad, scared, angry, or tired. Sheets representing 5 emoticons with associated feelings are shown to help the participant. If possible, the participant also indicates whether this feeling is little, normal, or intense. When it is too difficult for a participant to choose a specific emotion, the participant is asked whether he or she feels pleasant or nonpleasant.

Music workers will also complete a log report after each music session. The log report contains questions about that specific session: whether the session was performed according to the manual and how much time was spent on active music making.

#### Evaluation of Music Intervention

Participants in the music intervention and music workers will be asked to complete an evaluation questionnaire after the last music session. These questionnaires are designed to gain the participants’ personal views about the music intervention and their satisfaction with specific elements of it (eg, musical instruments and frequency of the sessions). The music workers will share their observations regarding behavioral, cognitive, and emotional changes within a participant during sessions.

After the last music session, caregivers will be asked to participate in a semistructured interview. This interview will focus on observed changes in the participants’ behavior and the experiences of caregivers with this music intervention study. Topics that will be covered are related to acceptability or satisfaction, reach, and context. The interviews will be recorded with the permission of the respondents and transcribed.

#### Follow-Up Evaluation

The participants in the music intervention and caregivers from participants in the music intervention will answer a few questions about the long-term use of musical activities after the last music session. For participants, this questionnaire will be conducted at T3, and for caregivers, it will be conducted 6 months after the last session.

### Sample Size Calculation

The sample size is calculated using G*Power software (version 3.1.9.7) [66]. The sample size calculation is based on a small effect size of 0.2, a statistical power of 0.95, and a type I error probability of α=.05, which results in an unadjusted sample size of 67 participants. Because cluster randomization negatively affects the power, the sample size needs to be adjusted. With the average cluster size of 2 participants per residential facility and allowing an intraclass correlation of 0.2, the adjusted sample size is 80. To allow for a dropout rate of 20% over the course of the study, we will aim at recruiting a total of at least 96 participants.

### Data Analysis

Participant characteristics will be compared between the group receiving the individual music intervention and the group receiving CAU. Further analyses will be adjusted for any potential significant differences. The primary analyses will be undertaken on an intention-to-treat basis, and 2-sided tests will be applied at a 5% α level. In addition, per-protocol analyses will be conducted based on the sample of participants who adequately adhered to the intervention protocol by completing at least 12 of the 16 music sessions (ie, 75%), which is similar to 67% and 80% of 2 other intervention studies among adults with mild to moderate ID [67,68]. In addition, by setting the threshold at 12 sessions, we know for sure that all intervention participants followed at least 1 or 2 sessions with a focus on emotions and EF.

To minimize the number of analyses on outcome domains, the 4 different EF measures will be converted into *z* scores and, according to factor analysis, summed up into an EF domain if possible. Subsequently, to test differences in changes from baseline to follow-up between the 2 conditions, a linear mixed model analysis for each treatment variable will be used. Within each analysis, only time and the interaction between the treatment variable and time will be used [69], making it possible to adjust for the dependence of the repeated observations within the subjects. The effect of the intervention on the 2 follow-up measurements will be assessed. This analysis allows for participants with only a baseline measurement but with missing data at follow-up to be included. The normality of the residuals from the linear mixed model analysis will be visually inspected.

Secondary analyses will be performed as well. The effect of extra music interventions will be tested applying a Mann-Whitney *U* test for the between-group comparisons. The self-reported feelings before and after each music session will be compared using a Wilcoxon signed rank test.

Descriptive statistics will be used to summarize the quantitative data of the log reports, evaluation questionnaires, and the follow-up evaluation.

Qualitative data of log reports, evaluation questionnaires, follow-up evaluation, recruitment log, trial log, and semistructured interviews will be analyzed using thematic context analysis on multiple aspects of the intervention and the overall study process, including recruitment, retention, acceptability or satisfaction, maintenance, dose, fidelity, reach, adaptation, and context.

### Ethical Considerations

Participation in the study will be voluntary. Participants and their legal representatives can withdraw consent at any time during the intervention and have the right to demand the removal of the original data. Random allocation of participants to 1 of the 2 conditions is considered reasonable as no adverse effects are expected in any of the conditions. Inconveniences caused by the necessity to attend 2 or 3 music sessions per week are considered tolerable in view of the anticipated benefit for the participant receiving music sessions. Participants assigned to CAU will receive a considerable budget after the study has ended, which they can spend on musical activities or musical instruments to their own wishes. Ethical approval for this study was granted by the review board of the Faculty of Behavioral and Movement Sciences of the VU University Amsterdam (protocol VCWE-2021-081). The Medical Ethical Committee of the VU University Medical Center stated that the research was not subject to the Dutch Medical Research Involving Human Subjects Act. The trial has been registered in the International Clinical Trial Registry Platform (NL8482).

### Data Management

To ensure the accuracy of the data, all entered data will be triple-checked by different research assistants. Every participant will be assigned an anonymous code after their informed consent form is received to store and identify their trial data. The coding key and person-related data will be saved in a password-protected file in a restricted folder, which will only be accessible by the study-related researchers. All paper files will be kept in locked cabinets in the researcher’s office for the duration of the study, and digital files will be stored on a password-protected and secure system, which is also exclusively accessible by the study-related researchers.

## Results

Enrollment commenced in July 2021, and data collection ended in May 2023. The aim to recruit at least 96 participants has been achieved with a total of 97 participants, of whom 44 were randomized in the intervention group. Data will be analyzed after this study protocol has been accepted for publication.

## Discussion

In this paper, the design of an RCT is presented, which aims at exploring the effectiveness of and experiences with a music intervention for adults with ID and CB on various outcome measures, including CB, well-being, depression, anxiety, self-esteem, and EF. The first strength of this study is that it concerns a clustered RCT design with a sufficient sample size. The second strength is that the multisource data collection, including the use of self-report questionnaires and tests as well as proxy measures, improves the research quality. Third, because distal outcomes (measured over a longer period of time) and proximal outcomes (measured immediately after the intervention) will be used, immediate effects can be assessed too. Lastly, as qualitative data are collected alongside quantitative data, further insight into the process or mechanisms of change can be gained.

However, some limitations also have to be taken into consideration. First, blinding is not possible in the design because the nature of the intervention and the different questionnaires at the data collection points for both groups precludes blinding of the assignment for all parties involved. The absence of an active control condition might entail difficulties in ascribing possibly disclosed effects to power factors of the music intervention, such as level of attention [70]. Furthermore, there are several anticipated challenges, which are based on previous work conducting RCTs with adults with ID. These include challenges with recruiting adequate numbers, resistance to the use of control groups, assessing participants through gatekeepers (ie, the caregivers), and staff turnover that impacts the data collection [25]. The following steps to minimize attrition are considered in this study: (1) the research team follows a flexible participant-led approach to gathering all data—with contacts being at times suggested by participants and at the convenience of the facility of the participant; (2) the incentive to complete all questionnaires and tests is increased with the compliment card that participants receive after every data collection measurement; (3) caregivers will receive multiple reminders, either live, via mail, or telephone, to return the questionnaires on time; and (4) the combination of collecting data from both participants and caregivers reduces the burden for them and diminishes the impact of participant dropout or staff turnover.

In sum, more evidence-based interventions are needed to help improve the mental well-being of this population. It is important to not only fill this evidence-base gap with the outcomes of the ID RCTs but also share the practical experiences conducting trials through the reporting of process evaluations. Therefore, it is expected that this study will provide insight into the effectiveness and experiences of an individual music intervention for adults with ID and CB. If the intervention proves to be effective, broad-scale implementation in the care for people with ID can be considered and results can guide further music intervention guidelines and enrich the work environment of music workers engaging in supporting people with ID.

## Abbreviations

ABC: Aberrant Behavior Checklist
CAU: care as usual
CB: challenging behavior
EF: executive functioning
ID: intellectual disabilities
PWI-ID: Personal Well-being Index—Intellectual Disability
RCT: randomized controlled trial
RE-AIM: Reach, Effectiveness, Adoption, Implementation, and Maintenance
QoL: quality of life
